# Acoustic Signaling by Singing Humpback Whales (*Megaptera novaeangliae*): What Role Does Reverberation Play?

**DOI:** 10.1371/journal.pone.0167277

**Published:** 2016-12-01

**Authors:** Eduardo Mercado

**Affiliations:** 1 Department of Psychology, University at Buffalo, The State University of New York, Buffalo, New York, United States of America; 2 Evolution, Ecology, and Behavior Program, University at Buffalo, The State University of New York, Buffalo, New York, United States of America; Institute of Deep-sea Science and Engineering, Chinese Academy of Sciences, CHINA

## Abstract

When humpback whales (*Megaptera novaeangliae*) sing in coastal waters, the units they produce can generate reverberation. Traditionally, such reverberant acoustic energy has been viewed as an incidental side-effect of high-amplitude, long-distance, sound transmission in the ocean. An alternative possibility, however, is that reverberation actually contributes to the structure and function of songs. In the current study, this possibility was assessed by analyzing reverberation generated by humpback whale song units, as well as the spectral structure of unit sequences, produced by singers from different regions. Acoustical analyses revealed that: (1) a subset of units within songs generated narrowband reverberant energy that in some cases persisted for periods longer than the interval between units; (2) these highly reverberant units were regularly repeated throughout the production of songs; and (3) units occurring before and after these units often contained spectral energy peaks at non-overlapping, adjacent frequencies that were systematically related to the bands of reverberant energy generated by the units. These findings strongly suggest that some singing humpback whales not only produce sounds conducive to long-duration reverberation, but also may sequentially structure songs to avoid spectral overlap between units and ongoing reverberation. Singer-generated reverberant energy that is received simultaneously with directly transmitted song units can potentially provide listening whales with spatial cues that may enable them to more accurately determine a singer’s position.

## Introduction

Sound transmission in natural environments can be strongly affected by the qualities of ambient noise as well as the geometry of the channels within which signals are broadcast [1–3]. Animals have developed a variety of mechanisms for overcoming such constraints, including adaptive vocal control [4], strategic selection of the time and place of sound production [5], and the development of specialized structures that enhance hearing sensitivity for specific acoustic features [6]. Animals that produce sounds in enclosed spaces are known to adjust their sound production based on environmental conditions [7], and some birds may adjust their positions in ways that increase the distance that their vocalizations propagate [5, 8]. When attempting to understand how a particular animal makes use of sound, it is thus important to keep in mind how the channel within which sounds are generated may affect sound production and use.

Environmental constraints are particularly critical to the functionality of acoustic communication signals transmitted over long distances [9]. In certain conditions, such as when sounds are transmitted through dense vegetation or in shallow water, sounds can undergo multiple reflections as they propagate, leading to reverberation that persists beyond the duration of the original signal [10]. Reverberation is especially evident in ocean environments where certain species of baleen whales produce high-amplitude sounds that can travel over distances greater than 10 km [11, 12]. The extent to which animals living in highly reverberant habitats account for the possible effects of reverberation when transmitting sounds over long distances (either actively or through evolved traits) remains unclear.

Sounds that vary in frequency over time tend to become distorted by reverberation [1, 13], degrading the ability of listeners to identify detailed acoustic features of received signals. Although reverberation-related distortion obscures the details of transmitted signals, it provides listeners with clues to the position of the sender because signal distortion varies as a function of the distance a sound has travelled [13]. In contrast, tonal sounds that contain energy focused within a narrow frequency band potentially can benefit from reverberation during long-range transmission, because the reverberated acoustic energy tends to reinforce the transmitted signal, leading to longer and louder received signals at farther distances [14, 15]. However, such sounds may provide less reliable information about the position of the individual producing the sounds, because the sound amplitude received by listeners will be similar at many ranges, and because the kinds of distortion-related cues to source distance associated with frequency-modulated sounds will not be available [16]. Consequently, senders transmitting narrowband sounds in reverberant environments often face a tradeoff between maximizing propagation range and making it easier for listeners to determine their location [14, 17].

Tonal sounds with relatively little frequency modulation are a common element of the long-distance signals produced by mammals and birds [18–20]. For example, humpback whales (*Megaptera novaeangliae*) produce sequences (called songs) containing a variety of tonal sounds that may travel several kilometers, and that are thought to play a key role in their mating systems [21, 22] Given that humpback whales generally do not maintain territories and travel long distances each year [23], the utility of songs is contingent upon the ability of listening whales to determine the locations of singers. To date, there have been few proposals about how listening humpback whales might extract any spatial information from songs that have travelled long distances [12, 24], and no consideration of how reverberation in the habitats where humpback whales sing might affect song structure or function.

The overall goal of the current study was to determine whether humpback whale songs are structured in ways that affect either song-generated reverberation levels or the localizability of singers. Humpback whales often sing for hours at a time in coastal waters [25–29]. Individual sounds within humpback whale songs (called units) travel multiple kilometers and are thought to affect the actions of other whales located at these long distances [22]. Units within songs typically reflect from the ocean surface and bottom multiple times as they propagate out from a singer [3], leading to complex variations in received signals as a function of time, distance, and signal frequency [30, 31]. To evaluate how sounds produced by singing humpback whales reverberate, in the current study the spectral properties of units and subsequent reverberation were analyzed. Additionally, the spectral profiles of sequential units were compared to determine whether the order of units produced by singing humpback whales might be related to the effects of reverberation on signal transmission.

## Materials and Methods

### Recordings

Humpback whales singing in a particular region generally produce songs with comparable structural features within a given year [28, 32–34]. However, the acoustic features present within humpback whale songs (e.g., the prevalence of different sound patterns, rate of sound production, use of different frequencies, and unit qualities) can vary considerably both within and across individuals, populations, and years [35, 36]. The sample of recordings analyzed in the current study constitutes a nonprobability sample of convenience (i.e., songs were sampled based on their availability and quality rather than randomly, and thus are not suitable for drawing statistical inferences about the whole population of singing humpbacks), including extended segments of a few high quality recordings of song sessions collected by various investigators, using different recording approaches, from multiple years and populations. The structural features of songs present within this sample are consistent with numerous published descriptions and spectrographic illustrations of humpback whale songs [28, 29, 32, 34, 35, 37–40]. Nevertheless, the current analyses should be viewed as case studies rather than as representing what singing humpback whales typically do. The sample of recordings analyzed here was chosen to establish how units can reverberate and to demonstrate that features of songs related to reverberation are not idiosyncratic to songs produced in a single locale or year.

Six archival recordings were used: two recorded in waters off of Maui, two from singers in the Indian Ocean, one from a singer near Puerto Rico, and one recorded in the northern Columbian Pacific Ocean. None of the recordings was collected specifically to investigate song-generated reverberation, and none was chosen based on the degree of reverberation evident within the recording. All recordings featured a single singer in close proximity to one or more hydrophones. Neither singer depths nor hydrophone depths were explicitly measured for any recording. However, singers typically are found at depths of 15–25 m [41] and hydrophones suspended from boats were positioned at depths less than 25 m.

The recordings made near Maui were collected in 2002 (by M. Lammers), and in 2007 (by D. Rothenberg). The 2002 recording, lasting 16 min, was collected in the Auau Channel between the islands of Maui, Lanai, Kahoolawe, and Molokai. It was made by a diver using a Sony digital audio tape recorder encased in an underwater housing at close range to the singer [41, 42]. The 2007 recording (12 min) was made off the coast of Maui from a boat using two Cetacean Research SQ26-08 hydrophones connected to a Sony MZ- M10 Hi-MD Minidisc Recorder, and stored as uncompressed PCM audio sampled at 44.1 kHz [43, 44]. The bathymetry and bottom composition at the specific locations of these recordings is unknown, but singing whales are most commonly found in waters surrounding Maui that are less than 200 m deep [45], where the bottom usually consists of silty sand and clay with intermittent outcrops of coral and rocks [3].

The Indian Ocean recordings were collected in 2007 (by O. Adam), and in 2013 (by the Darewin group). The 2007 recording, lasting 10 min, was made near the Sainte Marie Island Channel, where the water depth varies between 30 and 40 m [44, 46]. Recordings were collected from a small boat using a COLMAR Italia GP0280 hydrophone connected to its amplifier and a HD-P2 TASCAM recorder (sampling frequency = 44.1 kHz). The Réunion Island recording (26 min; http://sabiod.univ-tln.fr/nips4b/media/NIPS4B_Humpback_Darewin_LaReunion_Jul_03_2013-001_26min.wav) was collected by a diver in close proximity to a singer using a Zoom digital audio recorder (44.1 kHz sampling rate) encased in an underwater housing [47]. The water depth was < 80 m and the bottom composition was not determined.

The recording of a whale singing off the coast of Rincon, Puerto Rico, collected in 2009 by J. Schneider (38 min) was made using a hydrophone (Cetacean Research C10; 0.25–25 kHz flat frequency range, ±3 dB) suspended (~8 m depth) from a small raft tethered to a free-floating boat. The hydrophone was connected to a pre-amplifier (Cetacean Research Model SS03), which fed into a digital recorder (Sony MD Walkman Mz-NH900, recording in.wav format), sampling at a rate of 44.1 kHz. Bathymetry in the area where the recording was made involves a shallow-water shelf (< 100 m deep), just off the coast, that borders a rapid drop off to more than 600 m deep [48, 49].

The Colombian recording (32 min, made by C. Perazio) was collected in coastal waters of the Gulf of Tribuga [50] using a single SQ26-08 hydrophone suspended from a small boat, connected to a 24-bit Zoom H1 digital recorder (96 kHz sampling rate). Bathymetry in this region consists of an inclined shelf that reaches a depth of 300 m a few kilometers from the coast. The specific water depth and bottom properties associated with this recording are unknown.

The populations of humpback whales that sing in the Caribbean, Pacific Ocean, and Indian Ocean do not overlap; past analyses of song structure suggest that there should be little structural overlap in songs from these locations [26, 51].

### Selection and Analysis of Unit Features

Raven Pro 1.4 was used to automatically detect units within recordings and to collect measurements of their acoustic features. Units were isolated using band limited energy detection, with frequency ranges customized based on information from spectrographic images. Automatic detections of units were evaluated through visual inspection. Manual selections were made for undetected units and selections were manually adjusted when automatic detection overestimated or underestimated the duration of a unit. Several acoustic measurements were automatically collected from each unit including start and stop times, frequency with peak energy, and full bandwidth root mean square amplitude. These measurements made it possible to assess how consistently singers repeated units within songs.

Spectra and spectrograms were calculated for all units (FFT size = 4096 for recordings sampled at 44.1 kHz, or 8600 for the recording sampled at 96 kHz; Hann window, 50% overlap, providing a frequency resolution of ~16 Hz). Silent intervals between sounds were visually inspected in spectrographic representations generated by Raven to identify sound elements that produced reverberation and to estimate the duration and consistency of reverberation. Brightness and contrast settings were adjusted to accentuate any acoustic energy within the silent intervals between units. Similarly, the frequency range displayed within spectrograms was adjusted to emphasize ranges where reverberation was evident.

Repeated sequences of sounds within songs (corresponding to phrases or subphrases) were subjectively identified to determine the period at which singers repeated these sequences. Units were extracted from each recording and units with comparable acoustic features and positions within sound patterns were combined together into.wav files (i.e., with silent intervals and other sounds removed). These files were imported into Matlab (Ver 7.12.0 R2011a) and analyzed using the *pwelch* function, which calculates an estimate of the power spectral density of a waveform (FFT size = 8192 for recordings sampled at 44.1 kHz, or 17200 for the recording sampled at 96 kHz, providing a frequency resolution of ~ 8 Hz). Differences in the frequency content of sequential units were measured both in terms of the absolute frequency difference and the ratio of the peak frequencies. To evaluate the relationship between the frequency content of consecutive units, first, the waveforms for all similar units produced within all instances of a particular sound pattern were concatenated into a single continuous waveform. Then, the spectrum for this set of units was calculated and used to identify spectral peaks. The same procedure was performed for units that were immediately subsequent to those included within the initial combined spectrum. Overlap between the spectra from consecutive units was analyzed both subjectively, through visual inspection, and quantitatively, by calculating the ratios of spectral peaks. These measures provided a way to assess overlap in frequency content within predictable sequences of units, as well as the variability of frequency content within repeated sound patterns.

## Results

### “Tails” of Reverberant Energy within Songs

**Fig 1:**
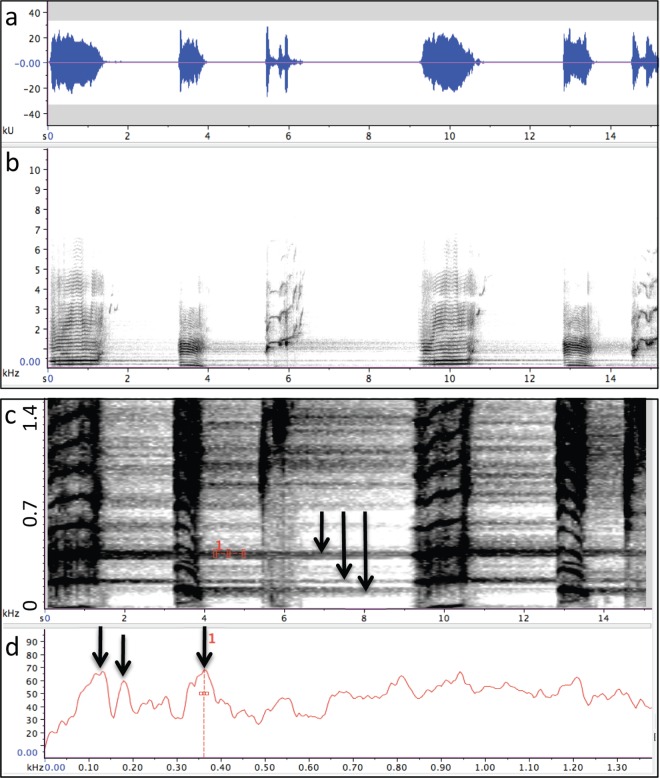
Reverberation generated by humpback whale song units. (a) Amplitude measures of units from a humpback whale song recorded off the coast of Maui in 2007 with a high signal-to-noise ratio (~48 dB), give the misimpression that little is happening acoustically during the intervals between units. (b) A spectrographic representation (FFT = 2048; Hann window; 95% overlap) that emphasizes frequency contours and harmonics of units, such as is commonly used to classify song phrases, shows reverberation as hazy bands between units that may appear similar to background noise and that are much less salient than units. (c) Reducing the frequency range and adjusting the brightness and contrast settings of the spectrogram shown in (b) reveals prominent bands of reverberation (highlighted with arrows) that persist long after each unit is produced. (d) a spectral analysis (FFT = 4096; Hann window; 50% overlap) of the interval of “silence” following the second unit in this example shows that the peak frequency of narrowband reverberant energy generated by the first unit (centered near 360 Hz) is ~40 dB above ambient noise levels more than 3 s after the unit has ended. Additionally, this spectrum shows that a reverberant “tail” generated by the second unit (centered near 130 Hz) falls just below a tail generated by the first unit (180 Hz; ratio = 1.3), with no spectral overlap.

Unit-generated reverberation was evident to varying extents in all of the recordings analyzed. However, reverberation was not consistently associated with all of the units within songs and was not evenly distributed across the full range of frequencies present within songs. The predominant forms of reverberation that were observed consisted of diffuse acoustic energy spread across a relatively wide band of frequencies and/or narrow bands of reverberation focused at one or two frequencies within a unit (Fig 1). Reverberated energy often persisted for several seconds. Although reverberation was usually visible for most spectrographic parameter settings, spectral analyses of intervals between units provided the clearest indications of how different units reverberated (Fig 1).

**Fig 2:**
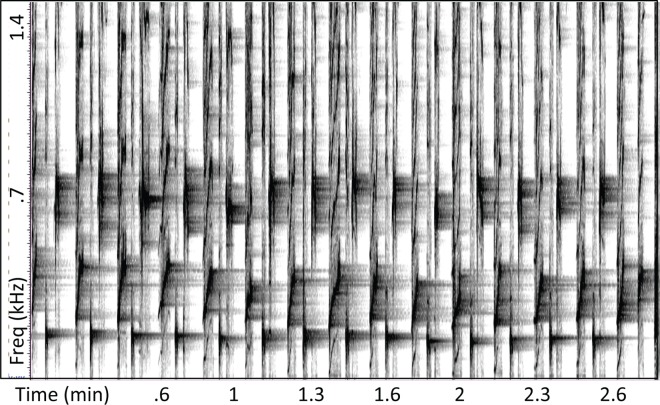
Reverberation generated by drone units. A subset of units (vertical bands) generated at regular intervals, produces a narrow band of reverberant acoustic energy (horizontal band centered at 165 Hz) that persists until just before the unit is repeated; these *drone units* typically occurred in multiple different sound patterns within a song. Note that the two units following the drone unit in the 3-unit pattern shown here also reverberate, but across a broader range of frequencies and less consistently. (2007 Maui recording; FFT = 8192, Hann, 50% overlap).

In two recordings (from Réunion Island and Maui), a subset of units generated narrow bands of reverberation that persisted until the singer repeated the same type of unit, such that reverberant bands overlapped with intervening units (Figs 1 and 2). In these cases, acoustic energy at a particular frequency persisted for minutes, with each repeated unit periodically boosting the spectral energy at that frequency (Fig 2). Units with these reverberant properties were not limited to a single segment of song, often recurring across multiple different themes. In music, the repeated or sustained production of a note throughout most of a piece is called a *drone*. Following this usage, units within humpback whale songs that were regularly produced in a highly consistent spectral and temporal manner within multiple sound patterns are hereafter referred to as *drone units*. Drone units were present in all six recordings analyzed; Table 1 summarizes their acoustic properties.

**Table 1:** Acoustic properties of drone units. Mean (standard deviation) measures of frequency with peak energy (Peak), unit duration (Dur) and period of drone unit repetition (Period) for all recordings.

	Drone Unit 1				Drone Unit 2			
Recording	*n*	Peak (Hz)	Dur (s)	Period (s)	*n*	Peak (Hz)	Dur (s)	Period (s)
Réunion Island	210	.47 (.4)	0.9 (.2)	7 (.7)				
Madagascar	57	.45 (.3)	1.6 (.9)	7.6 (3)				
Puerto Rico	102	.46 (.1)	1.8 (.4)	16 (2)	31	.25 (.2)	1.3 (.2)	17 (3)
Colombia	183	.1 (.1)	1.1 (.6)	—	68	.26 (.1)	1.6 (.4)	—
Maui (2007)	49	.34 (.2)	1.7 (.7)	11 (2)				
Maui (2002)	37	.55 (.03)	1.5 (.4)	—	126	.88 (.8)	0.8 (.2)	—

A dash (—) indicates that drone units were interspersed with other units rather than produced with a fixed period.

**Fig 3:**
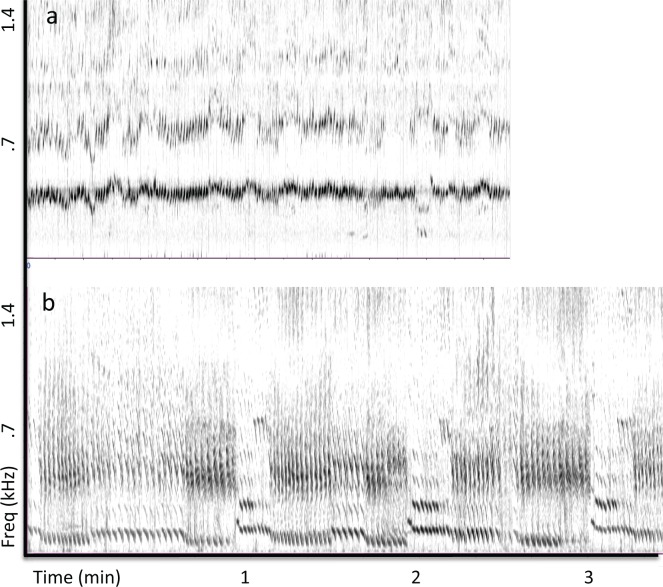
Variability of drone units across repetitions. (a) Spectrogram (FFT = 4096, Hann window, 50% overlap) of 210 consecutive drone units (with following units/silences removed) recorded near Réunion Island shows that the frequency content of these units remained highly stable throughout the 26 min recording. (b) Spectrogram of 132 consecutive drone units recorded off the coast of Puerto Rico shows subtle shifts in the spectral content of these units over time, with energy consistently focused near 130 and 500 Hz.

**Fig 4:**
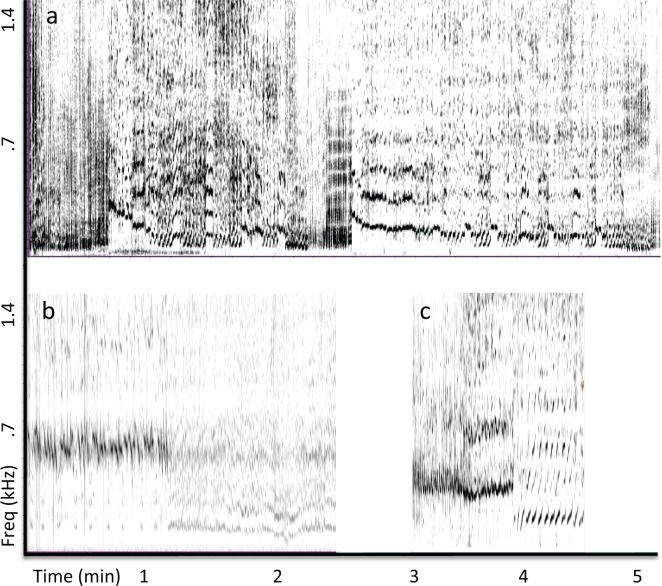
Variability of drone units across repetitions. (a) Spectrogram (FFT = 17200, Hann window, 50% overlap) of 251 consecutive drone units (sans following units/silences) recorded near Colombia shows gradual shifts in spectral content with repetition, as well as more discrete shifts in spectral content. (b) Spectrogram (FFT = 8192, Hann window, 50% overlap) of 163 drone units recorded off the coast of Maui in 2002 shows a large shift in fundamental frequency. (c) A similar shift in drone unit frequency content was evident in the Madagascar recording (57 units).

Drone units in the recording from Réunion Island matched the frequency of a reverberant band (~400 Hz) that was present throughout the recording. Drone units from this recording showed highly stable spectral and temporal features, repeating every 7 s on average (Table 1; Fig 3A). The Madagascar recording (from the same population of whales that visit Réunion Island) contained drone units with similar spectral and temporal properties to those observed subsequently at Réunion Island (Table 1; Fig 4C), but was acoustically more variable with less evidence of sustained reverberation. Whereas drone units were present throughout the Réunion Island recording (i.e., occurring in all identified sound patterns), they were less prevalent in the Madagascar recording, appearing in only three of the six sound patterns identified.

The recording of song from Puerto Rican waters revealed relatively little evidence of prolonged reverberation at specific frequencies. When reverberation from units was evident, it was often shorter in duration than inter-unit intervals. Nevertheless, periodically produced drone units with spectral energy focused in a narrow band were found throughout this recording (i.e., in all identified sound patterns), as in the Réunion Island recording. Drone units in the Puerto Rican recording were more variable in frequency content (Fig 3B), lower in fundamental frequency (80–150 Hz), and produced with a longer period (~16 s, see Table 1), than those in the Indian Ocean recordings. Although reverberation was not continuously present in the Puerto Rican recording, continuous bands of reverberant energy that were focused at frequencies matching those of drone units, and lasting more than 10 s, appeared intermittently within the recording. The spectral properties of drone units appeared to alternate between two bands (Fig 3B; Table 1)

Reverberation within the recording from Colombia was comparable to that present in the Puerto Rican recording, consisting mainly of energy focused within narrow bands that matched the peak frequencies of drone units and that persisted for a few seconds. However, a notable difference in the drone units produced near Colombia was that they showed a gradual, cyclical shift in frequency content over time, rather than remaining focused at one or two frequencies (Fig 4A). Additionally, drone units in the Colombian recording were not produced at a single fixed rate, but were interspersed with other units in an alternating pattern.

The Maui recording made in 2002 showed the least evidence of unit-generated reverberation. When reverberant energy was present, it generally lasted less than a second and was not clearly focused within a narrow band. Drone units were alternated with other units, as in the Colombian recording, rather than being repeated at a fixed rate. Drone units from the 2002 Maui recording fell into two acoustically distinctive categories, which in some cases were mixed within a single sound pattern (Fig 4B). The only other recording that showed such a large spectral difference between drone units was the Madagascar recording (Fig 4C).

Reverberation was most evident in the 2007 recording from Maui (Fig 2), with energy again focused in narrow bands that matched the spectral peaks of drone units. As illustrated in Fig 1, narrowband reverberant energy generated by drone units in this recording sometimes persisted until the next drone unit was produced (i.e., 9–11 s), could occur at more than one frequency, and could be greater at frequencies other than the fundamental frequency.

### Spectral Interleaving of Unit Sequences

**Fig 5:**
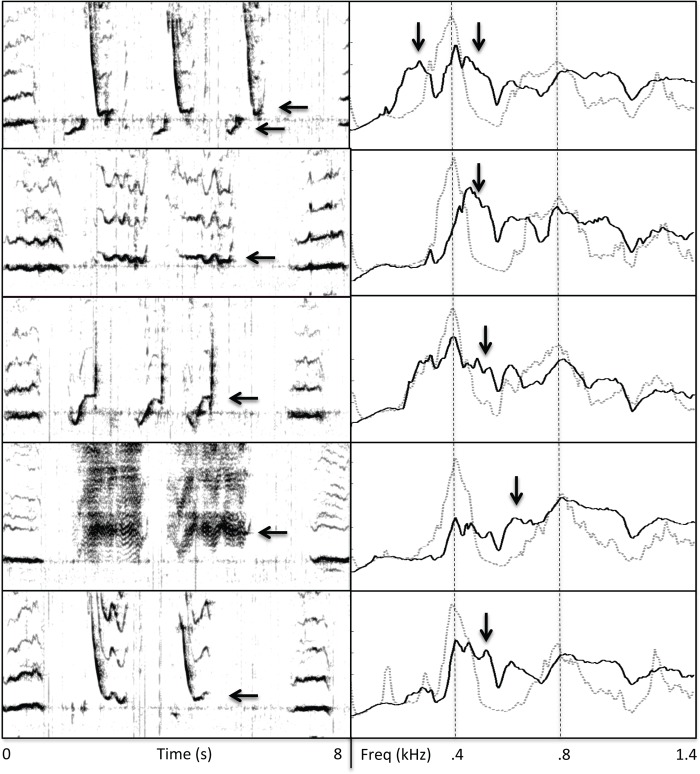
Examples of all repeated sound patterns sung by a humpback whale near Réunion Island. (left) Spectrograms show variations in the number and features of units following drone units (FFT = 4096; y-axis = 0–1.4 kHz). Arrows show how frequencies with peak energy content straddle a reverberant band that matches the fundamental frequency of the drone units. (right) Spectra (FFT = 8192) calculated across all instances of drone units (dotted gray lines) and all following units (black lines) for each pattern type show that the distribution of spectral energy within following units spans regions surrounding frequencies with peak energy from drone units (vertical dashed lines); note, in particular the areas between the two spectra curves.

**Fig 6:**
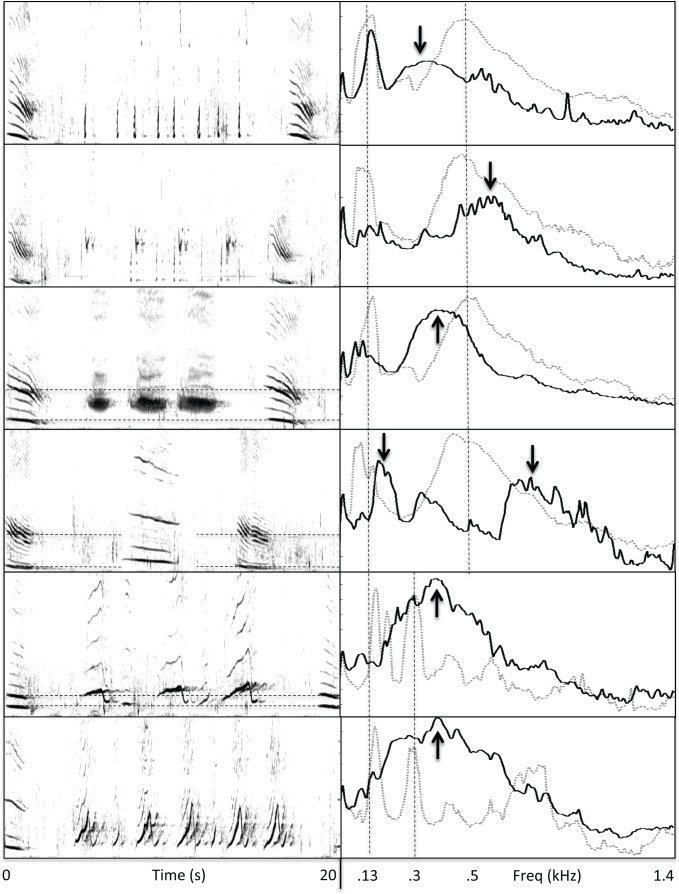
Examples of all repeated sound patterns sung off the coast of Puerto Rico. (left) Spectrograms show variations in the number and features of units following drone units (FFT = 4096; y-axis = 0–1.4 kHz). Dashed lines show how frequencies with peak energy in drone units are systematically related to, and even interdigitated with, the peak frequencies of following units. (right) Spectra (FFT = 8192) calculated across all instances of drone units (dotted gray lines) and all following units (black lines) for each pattern type show that the distribution of spectral energy within following units spans regions adjacent to frequencies with peak energy from drone units (vertical dashed lines); note, in particular the areas between the two spectra curves. Arrows show peak frequencies of following units that are adjacent to peak frequencies of drone units.

Visual inspection of spectrograms from recordings revealed that the frequency content of units immediately subsequent to drone units (referred to as *following units*) was often systematically related to the spectral properties of drone units. Specifically, following units often contained peak frequencies adjacent to the peak frequencies of the drone unit. This was especially evident when reverberant tails were present, because of the close spacing between reverberant bands from drone units and the spectral bands generated by following units (e.g., see Fig 1). Following units typically exhibited a broader range of frequency modulation than drone units, such that the frequencies where most energy was focused were not always evident from visual inspection of spectrograms. Fig 5 shows representative examples of following units at different stages in the song progression of the Réunion Island recording, and Fig 6 shows examples from the Puerto Rican recording. In both recordings, the spectra of following units contained peaks at frequencies just above or below the frequencies with peak energy in drone units for all of the sound patterns present within the recordings.

**Fig 7:**
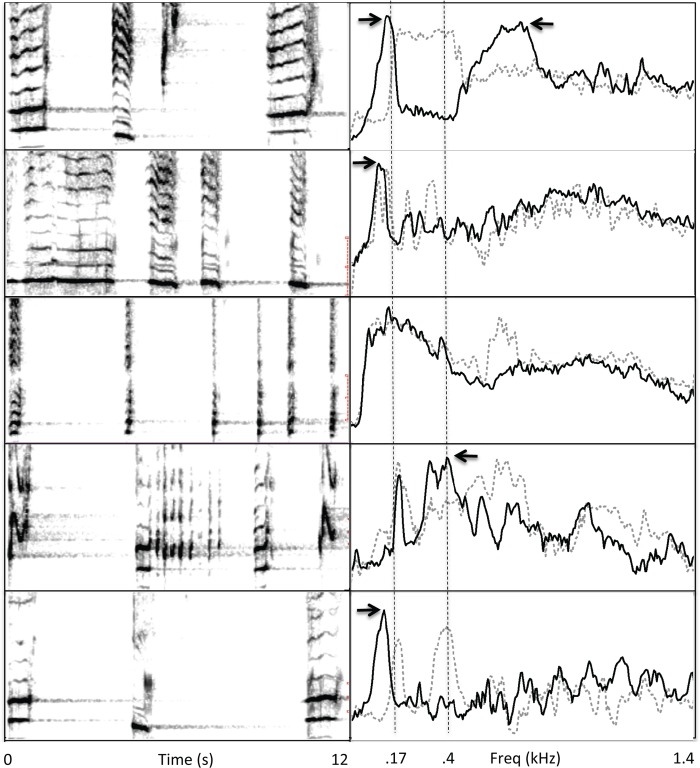
Examples of all repeated sound patterns sung off the coast of Maui (2007). (left) Spectrograms (FFT = 4096; y-axis = 0–1.4 kHz) show variations in the number and features of units following drone units (1^st^, 2^nd^, 4^th^, and 5^th^ images), as well as when drone units were not part of a pattern (3^rd^ row). (right) Spectra (FFT = 8192) calculated across all instances of drone units (dotted gray lines) and all following units (black lines) for each pattern type show that the distribution of spectral energy within following units spans regions adjacent to frequencies with peak energy from drone units. Arrows show peak frequencies of following units that are adjacent to peak frequencies of drone units. For the sound pattern without drone units, the spectrum of the longest duration unit in the pattern was used as a basis for comparison.

**Fig 8:**
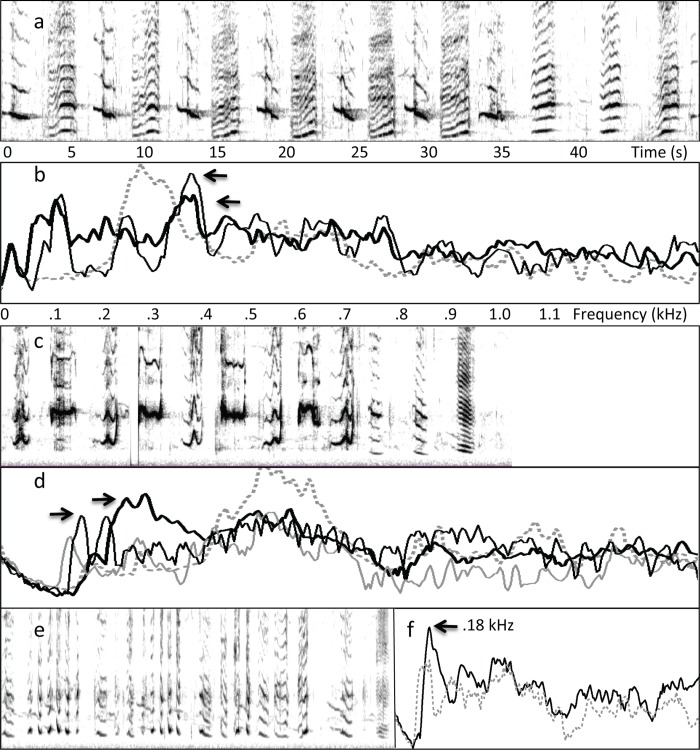
Examples of spectral interleaving involving alternating units. (a) Spectrogram (FFT = 8600; y-axis = 0–1.4 kHz) of a Colombian song shows repeated alternations of a drone unit and following unit. (b) Spectra (FFT = 17200) calculated across all instances of drone units (dotted gray line) within the pattern shown in (a), and all following units (black lines), show that the spectral peaks of following units (381 and 387 Hz) border those of drone units (281 Hz; ratio = 1.4); the thinner solid line is the spectrum of the last three units. (c) Spectrogram (FFT = 4096; y-axis = 0–1.4 kHz) from the Maui 2002 recording shows similarly alternating units. (d) Spectra (FFT = 8192) calculated across all instances of drone units (dotted gray line and solid gray line) within the pattern shown in (c) and all following units (black lines) show that the spectral peaks of following units (161 and 291 Hz) span regions adjacent to those of drone units (peaks of 140 and 522 Hz); the thinner lines are spectra of the last three units (gray = 1^st^ and 2^nd^, black = 3^rd^). (e) Spectrogram of a second complex pattern from the Maui 2002 recording showing mixing of drone units with following units. (f) Spectra of drone (156 Hz peak) and following units (178 Hz peak; ratio = 1.1) show tight spectral interleaving within the pattern. x-axis time/frequency scales apply to all spectrograms/spectra other than (f).

Similar spectral interleaving was also evident in the 2007 recording from Maui (Fig 7) and in the recording from Madagascar, for all patterns that included drone units (4 of 5 sound patterns in the Maui recording and 3 of 6 in the Madagascar recording). When drone units were not present in a sound pattern, units with similar spectra were often repeated (Fig 7, third row). The distribution of drone units present within the 2002 recording from Maui and the 2013 recording from Colombia was more complex (Fig 8). Specifically, drone units tended to alternate with following units within sound patterns. Units following each drone unit showed evidence of spectral interleaving, even in these more complex patterns (Fig 8).

Spectral relationships were quantified for 207 drone units from the Réunion Island recording, 132 drone units from the Puerto Rican recording, and 49 drone units from the Maui 2007 recording. Table 2 summarizes the relationships revealed through these comparisons. Although the absolute frequencies and the period of drone units varied across the six recordings analyzed, the spectral relationships of following units to drone units were surprisingly consistent across recordings.

**Table 2:** Relationships between frequencies with peak energy across sequences of units. Maxima of spectra for lower (Peak 1) and higher (Peak 2) frequency peaks measured from all units within different pattern types (Figs 5–7). Spectra from all drone units used within a pattern type were compared with spectra from associated following units. Ratios were calculated by dividing the higher frequency of a pair by the lower frequency.

	Drone Unit		Following Units		Frequency Ratios	
Sound Pattern	Peak 1 (Hz)	Peak 2 (Hz)	Peak 1 (Hz)	Peak 2 (Hz)	Ratio 1	Ratio 2
**Réunion Island**						
Pattern 1 (*n* = 101)	398	807	420	280	1.06	1.42
Pattern 2 (*n* = 19)	409	818	474	813	1.16	1.01
Pattern 3 (*n* = 21)	398	818	404	834	1.02	1.02
Pattern 4 (*n* = 38)	415	829	415	823	1.0	1.01
Pattern 5 (*n* = 28)	398	813	415	840	1.04	1.03
MEAN (STDEV)					1.06 (.06)	1.1 (.18)
**Puerto Rico**						
Pattern 1 (*n* = 34)	135	501	129	355	1.05	1.41
Pattern 2 (*n* = 7)	92	490	167	565	1.82	1.15
Pattern 3 (*n* = 24)	135	517	97	409	1.39	1.26
Pattern 4 (*n* = 19)	86	463	162	770	1.88	1.66
Pattern 5 (*n* = 32)	145	302	—	388	—	1.28
Pattern 6 (*n* = 16)	145	301	—	398	—	1.32
MEAN (STDEV)					1.54 (.39)	1.35 (.18)
**Maui (2007)**						
Pattern 1 (*n* = 26)	205*	420*	151	695	1.36	1.65
Pattern 2 (*n* = 4)	151	334	113	—	1.34	—
Pattern 3 (*n* = 12)	*608*	*107*	*156*	*178*	*3*.*90*	*1*.*66*
Pattern 4 (*n* = 2)	*603*	*193*	*393*	*199*	*1*.*53*	*1*.*03*
Pattern 5 (*n* = 11)	393	194	—	135	—	1.44

* indicates there was not a clear spectral peak; italics indicate that the pattern did not contain drone units, in which case measures collected from the unit of longest duration were substituted.

## Discussion

The analyses conducted in this study revealed several intriguing features of humpback whale songs that have not previously been noted in the literature. First, a subset of units within songs were found to be capable of generating persistent reverberant energy, sometimes lasting more than five times the duration of the reverberating units. Second, the reverberant energy generated by these units was often focused within one or two narrow frequency bands, despite the fact that the spectral energy within the units typically spanned several octaves. Third, units that generated such reverberant bands (described here as drone units) typically were repeated with high consistency throughout a song session, remaining spectrally and sometimes temporally stereotyped, even when a singer switched between themes. Fourth, the frequency content of units surrounding drone units was often adjacent to the peak frequencies of drone units, such that reverberant bands from such units showed minimal spectral overlap with subsequent units. Collectively, these acoustic features strongly suggest that reverberation generated by humpback whale songs is not simply an inadvertent side-effect of highly energetic sound production underwater, but instead may play a key role in determining the structure of humpback whale songs and might potentially affect how they function.

Reverberation generated by singing humpback whales is often audible in recordings and even the earliest scientific descriptions of song structure included spectrograms showing evidence of reverberation from songs [28, 38]. Little scientific attention has been given to this aspect of song production, however. When reverberation produced by singing whales has been discussed, it has usually been cited as a possible source of signal distortion (e.g., [52]). Researchers have proposed that other baleen whales might use reverberation as a way to detect environmental features [53, 54], but to date there have been no investigations examining the propensity of different whale sounds to reverberate. Prior analyses of the structural features of humpback whale songs have failed to report the properties of drone units [28, 33, 34, 38, 39, 55] or of reverberation generated by such units, raising the question of why these properties were not identified earlier. Drone units may not have attracted attention in earlier acoustic analyses because: (1) their spectral features and repetition rates may vary across recordings; (2) spectrographic and aural analyses can obscure the ways in which drone units differ from following units; (3) the greater number and variety of units surrounding drone units makes following units more useful for identifying song phrases and theme transitions; (4) past approaches to analyzing humpback whale songs have emphasized patterns in the frequency contours of individual units, rather than the spectral energy within units or within intervals of silence between units; and (5) reverberation levels generated by nearby singers are lower than direct signal levels and therefore less visually salient in spectrograms configured to emphasize the frequency contours or harmonics of units (Fig 1).

Another reason that the reverberant properties of drone units may have been overlooked in previous studies is that high levels of reverberation are not evident in all recordings. For example, reverberation generated by drone units in the Puerto Rican and Maui 2002 recordings was much less prominent than reverberation generated by similar units in other recordings. Although the current analyses make it clear that drone units within songs can generate sustained bands of reverberant energy, they also show that this outcome is not inevitable. What then determines when drone units (or other units) will persistently reverberate? One major factor is the sound channel within which a song is produced [30, 56–58]. Explosions produced in coastal environments can generate reverberation lasting 30 s or more, mainly because of scattering of incident sound by bottom irregularities [55]. Little is known about how singers decide when and where to sing, but recent research suggests that bathymetric features are predictive of where singers are likely to be found [49]. In particular, singers are consistently found in shallow waters (< 200 m deep), over harder bottoms that are relatively flat [3, 59]. In Puerto Rico, singers tend to congregate near the edges of shelves [49]. Although both types of environments tend to be highly reverberant [30, 57], the extent to which songs generate sustained reverberant bands is likely to vary considerably as a function of a singer’s position as well as the acoustic features of constituent units within a song [3, 48].

Individual humpback whales are known to progressively change the structural qualities of their songs continuously throughout their lives, and to vary the time they spend producing particular sequential patterns of units, even within a single singing session [28, 32]. It is also well established that singers can produce units with a wide range of spectral characteristics [28, 41, 60]. Given this vocal flexibility, and the finding from the current study that singers can produce units that do not strongly reverberate, singers should be capable of avoiding producing high levels of reverberation. The finding that singers produce drone units that can generate long-lasting reverberation, and produce subsequent units in ways that avoid spectral overlap with ongoing reverberant energy, strongly suggest that reverberation plays an important role in song production.

The possible benefits singers may gain from including highly reverberant units within songs remain to be determined. Past work examining reverberation induced by bird songs has shown that distance-related variations in received reverberation can sometimes provide listening birds with cues about how far a song has traveled, thereby enabling listeners to judge the location of the singer [1, 13, 17, 61]. Producing reverberation that coincides with subsequent, spectrally adjacent units may similarly provide listening whales with useful cues to a singer’s position [62].

The current study was designed primarily to determine the extent to which individual sounds within humpback whale songs reverberate. The analyses were performed on a sample of convenience rather than selecting songs based on any evidence of reverberation within those recordings. Consequently, the current study is limited in what it can reveal about how commonly song production by humpback whales leads to sustained reverberation. Drone units were evident in all of the songs analyzed, but the prevalence and consistency of these units, as well as the levels of reverberation that they generated, differed across recordings. An important question for future research will be to determine how consistently individual singers use drone units within song sessions. The conditions that promote higher levels of unit reverberation should also be examined more closely.

Both simulation and experimental studies suggest that the frequencies that propagate best in areas where humpback whales sing can vary considerably as a function of environmental conditions [3, 48, 63]. In principle, singers might adjust the frequencies that they produce as a function of the habitat within which they are singing, thereby enhancing or suppressing the reverberation of drone units. Whether singers actively modulate the spectral content of their songs over time as a function of environmental conditions is not known. Studies that correlate spectral peaks within songs to surrounding bathymetric features (or other environmental factors) could clarify whether spectral variations in drone units reflect individual differences in song production or environmentally-dependent adjustments.

A related question concerns the role of ambient noise levels in song production, especially background sounds generated by other singers. Because reverberation decays over time, high ambient noise levels may decrease the ranges at which unit-generated reverberation remains detectable. Alternatively, singers could potentially increase the duration, intensity, or rate of drone units to counteract increases in noise levels. Future studies that relate the acoustic qualities of units to the acoustic conditions within which they were produced may shed light on the extent to which reverberation plays a role in song production when noise levels are high.

## Conclusions

The analyses reported here suggest that reverberation may play a more important role in humpback whale singing behavior than is generally assumed. If reverberation from song units impeded song function, then singers might be expected to produce units that were less prone to reverberating. Instead, at least some humpback whales appear to sing in ways that lead to sustained narrowband reverberation. Traditionally, bioacousticians have emphasized progressive variations in sound sequences (phrases and themes) when analyzing humpback whale songs, rather than analyzing acoustic variations in units or during the intervals between them. The current findings suggest that researchers should consider more closely the acoustic relationships between consecutive units (see also [59]), as well as the extent to which organizational features of songs reflect both these relationships and the reverberant properties of units.
